# The occurrence of asthma in an extensive-stage small-cell lung cancer patient after combination therapy with atezolizumab and anlotinib: a case report

**DOI:** 10.3389/fimmu.2024.1333850

**Published:** 2024-02-29

**Authors:** Wang Deng, Juan Chen, Xin-Yu Deng

**Affiliations:** ^1^ Department of Pulmonary and Critical Care Medicine, Second Affiliated Hospital of Chongqing Medical University, Chongqing, China; ^2^ Department of Pulmonary Medicine, Medical Research Center for Pulmonary and Critical Care Medicine, Chongqing, China

**Keywords:** atezolizumab, anlotinib, asthma, small-cell lung cancer, combined regimens

## Abstract

**Background:**

Extensive-stage small-cell lung cancer (ES-SCLC) is highly malignant, with early metastasis and high recurrence. Since therapeutic options are limited, ES-SCLC has a characteristically short survival period and extremely poor prognosis. A combination of immune checkpoint inhibitors (ICIs) and anti-angiogenic drugs can achieve promising efficacy and safety in patients with ES-SCLC as a second-line or subsequent treatment, extending survival to some extent. However, the clinical outcomes remain mostly unsatisfactory and are sometimes affected by treatment-related adverse events.

**Case presentation:**

A 57-year-old woman with ES-SCLC was administered a combination therapy of atezolizumab (a PD-L1 inhibitor) and anlotinib [an oral multi-targeted tyrosine kinase inhibitor (TKI)]. She survived for 22 months, with no disease progression during the 28 courses of therapy. Unexpectedly, despite having no history of asthma, the patient developed asthma while receiving this regimen. This is possibly related to T-cell activation and the tumor immune microenvironment, which induce allergic inflammation after PD-L1 blockade.

**Conclusions:**

This is the first report of an asthma-negative ES-SCLC patient who developed asthma after receiving atezolizumab plus anlotinib. Although this combination therapy may effectively extend survival in SCLC patients, asthmatic symptoms should be closely monitored.

## Introduction

Small-cell lung cancer (SCLC) is a highly malignant tumor with a poor prognosis, accounting for approximately 15% of all lung cancers, and is the leading cause of cancer-related deaths worldwide (1, 2). More than 50% of SCLC patients are diagnosed with extensive-stage (ES) disease (3). ES-SCLC is the most aggressive type of lung cancer, characterized by early metastasis, rapid proliferation rate, and high recurrence, with an average overall survival (OS) of only 2–4 months in its natural course (4, 5). After initial treatment with systemic chemotherapy and radiotherapy, current therapeutic strategies are limited to improving the long-term survival and reducing the mortality rate of ES-SCLC.

Comprehensive medical treatment should be a top priority for patients with ES-SCLC. Programmed death-ligand 1 (PD-L1) inhibitors and anti-angiogenic agents may represent new therapeutic strategies for ES-SCLC (6). The combination of immune checkpoint inhibitors (ICIs) with platinum-based chemotherapy has demonstrated sustained benefits in OS as a standard first-line option for current treatment (7). Anlotinib is a small-molecule tyrosine kinase inhibitor (TKI) that inhibits tumor neovascularization and negatively regulates tumor growth. Evidence indicates that anlotinib stimulates lymphocyte infiltration and migration in tumors, increasing the anticancer effects of PD-L1 inhibitors by reducing immunosuppression (8, 9). Several studies have reported promising efficacy and safety of the combination of ICIs and anlotinib as a second- or third-line treatment for ES-SCLC (10, 11).

Although immunotherapy offers some advantages over other anticancer regimens, its use is complicated by potentially lethal immune-related adverse events (irAEs), including skin toxicity (44%–68%), myocarditis (50%), colitis (10%–25%), nervous system toxicity (10%), and pneumonitis (9.6%) (12). However, to our knowledge, the development of bronchial asthma in patients with asthma-negative SCLC receiving immunotherapy has not yet been reported.

Herein, we report the case of an asthma-negative patient with ES-SCLC who experienced an asthma attack during treatment with atezolizumab in combination with anlotinib. Currently, the progression-free survival (PFS) of the patient has lasted for nearly 2 years. We have attempted to explain the reasons for this rare adverse effect.

## Case presentation

**Figure 1:**
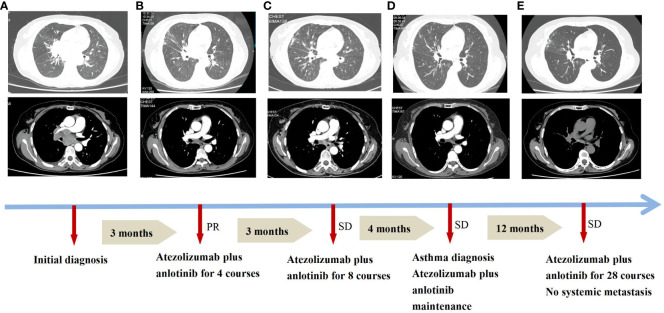
Treatment progress of atezolizumab plus anlotinib and asthma diagnosis in the patient. **(A)** Initial diagnosis. **(B)** Combined regimens for 4 courses. **(C)** Combined regimens for 8 courses. **(D)** Asthma diagnosis. **(E)** Combined regimens for 28 courses. PR, partial response; SD, stable disease. Efficacy was evaluated according to the Response Evaluation Criteria in Solid Tumours (RECIST).

**Figure 2:**
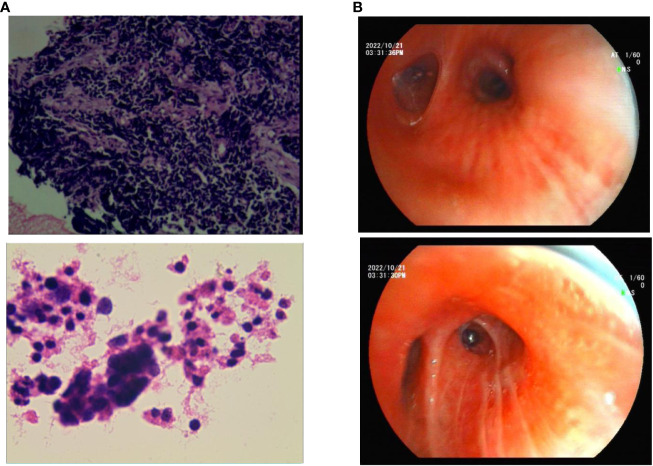
**(A)** Photograph of small-cell lung cancer. **(B)** Bronchoscopy during asthma diagnosis.

A 57-year-old Chinese woman with a 6-month history of cough and 1 week of dyspnea was admitted to our hospital on 25 January 2022. She was in good health with no history of asthma, allergies, or smoking, and no family history of hereditary disease, asthma, or tumors. She was retired from school teaching and lived alone with no pets in a nonsmoking environment. Initial physical examination showed normal results. Chest computed tomography (CT) revealed a central-type tumor in the right lung with invasion of the right pulmonary vein and right atrium and multiple lymph node metastases in the mediastinum and hilar regions (**Figure 1A**). Abdominal CT showed left adrenal gland and liver metastases, while no metastasis was detected on brain and systemic bone imaging. The electrocardiogram findings were normal. Laboratory findings indicated significant elevation of tumor markers, including carcinoembryonic antigen (CEA) and neuron-specific enolase (NSE). Routine blood tests and IgE, eosinophil, serum cTnl, CK-MB, and D-dimer levels were all within the normal ranges. Lung biopsy was performed using fiberoptic bronchoscopy and endobronchial ultrasonography. Histopathological analysis revealed TTF-1 (+), Syn (+), CgA (+), CD56 (+), CK7 (−), napsin A (−), CK5/6 (−), Ki-67 (40%+), CK (+), P40 (−), P63 (−), and PD-L1 <5% (**Figure 2A**). The patient was diagnosed with ES-SCLC (T4N2aM1c2, stage IVB).

**Figure 3:**
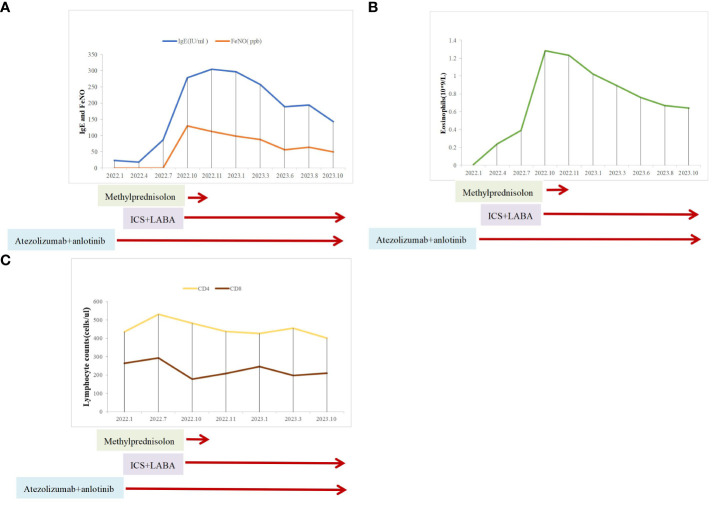
Clinical course of the patient after asthma diagnosis. **(A)** IgE and exhaled nitric oxide levels. **(B)** Eosinophil counts. **(C)** Lymphocyte counts.

Owing to her resistance to chemotherapy, the patient was administered atezolizumab in combination with anlotinib as the initial treatment strategy. After four courses of treatment, the tumor size decreased significantly (**Figure 1B**) and remained stable during subsequent treatment (**Figure 1C**). However, before the 12th course of treatment in October 2022, the patient experienced aggressive dyspnea (modified mMRC score ≥2) with wheezing rales in both lungs. She had no chest pain, hemoptysis, cough, sputum production, fever, urticaria, angioedema, abdominal pain, skin rash, or joint swelling and pain. Chest CT showed no change in tumor size and no pulmonary embolism (**Figure 1D**). Fiberoptic bronchoscopy revealed bronchial mucosal congestion, edema, and secretions, without obvious obstruction (**Figure 2B**). Bronchoalveolar lavage fluid tests for nucleic acid detection of respiratory pathogens (bacteria and viruses) and acid-fast bacilli and fungal smears yielded negative results. In turn, elevated eosinophil count (1.28 ∗ 10^9^/L) and IgE level (278 U/mL) were observed. The patient exhibited a positive response to a bronchodilation test, showing a 15% improvement in forced expiratory volume in 1 s (FEV1) and a 220-mL increase in the absolute FEV1 value in response to a beta-agonist. Pulmonary function test revealed 130 ppb of exhaled nitric oxide with no obstructive dysfunction. The patient was clinically diagnosed with asthma secondary to ICI treatment. She was started on systemic corticosteroids (methylprednisolone 40 mg/day for 5 days) and regular use of inhaled corticosteroids (ICS)/long-acting beta-agonists (LABA) (fluticasone propionate/salmeterol) and montelukast (10 mg/day). Her symptoms resolved with a decrease in eosinophil, IgE, and exhaled nitric oxide levels, along with normal lymphocyte counts during long-term therapy (**Figure 3**). Currently, her PFS has reached 22 months, with no systemic metastasis and a stable tumor status at the last follow-up (**Figure 1E**). In addition, reductions in serum CEA and NSE levels were recorded at the last follow-up. No tumor lysis syndrome or cytokine release syndrome was observed during treatment.

## Discussion

SCLC is a high-grade neuroendocrine cancer that is characterized by intensive invasiveness and rapid progression. Approximately two-thirds of SCLC patients are initially diagnosed with distant metastasis, mainly involving the liver, adrenal gland, brain, and bones (13). In the case of ES-SCLC, short survival and poor outcome significantly impact the quality of life, with a median OS of only 6–10 months when treated with ICIs plus chemotherapy (14) and a 5-year survival rate of less than 5% (15). Recent studies have shown that the combination of programmed death-1 (PD-1)/PD-L1 inhibitors and anti-angiogenic therapy can improve outcomes in ES-SCLC, with a PFS and OS of 3.4–7.5 and 8.2 months, respectively (10, 11). However, reports on long-term survival in ES-SCLC are relatively rare and may be attributed to factors such as better physical status, the absence of liver or brain metastases, sensitivity to platinum-based chemotherapy, and adherence to close follow-up (16, 17). Currently, this patient has achieved a survival of 22 months following atezolizumab in combination with anlotinib therapy. However, the specific mechanisms underlying the antitumor actions of PD-L1 inhibitors combined with anlotinib in ES-SCLC have not been sufficiently investigated.

Anlotinib is a multi-targeted TKI that exerts marked inhibitory effects on tumor angiogenesis by inhibiting vascular endothelial growth factor (VEGF), fibroblast growth factor receptor (FGFR), epidermal growth factor receptor (EGFR), platelet-derived growth factor receptor (PDGFR), and stem cell factor receptor (c-Kit) (18). In the clinical trial ALTER 1202, the SCLC group treated with anlotinib showed longer median PFS (4.1 *vs.* 0.7 months) and median OS (7.3 *vs.* 4.9 months) compared to the placebo group, reducing the risk of death by 47% (19). Anlotinib is currently the only antiangiogenic drug approved as third-line treatment for ES-SCLC in China. A recent study also showed that anlotinib was effective in SCLC as first-line maintenance therapy and second-line treatment, with no new anlotinib-related adverse reactions (20). However, the PFS or OS of anlotinib monotherapy or combination therapy is no more than 13 months, based on the current data.

Atezolizumab is a humanized anti-PD-L1-monoclonal antibody that regulates anticancer immunity by inhibiting PD-L1/PD-1 interactions (21). The IMpower133 trial concluded that atezolizumab plus chemotherapy significantly improved OS and PFS in ES-SCLS as first-line treatment (22). However, ICIs monotherapy did not demonstrate clinical benefits in terms of OS for ES-SCLC as a second-line or subsequent therapeutic option (23). Tumorigenesis can lead to a reduction in dendritic cells (DCs) by impairing antigen presentation and preventing T-cell activation, resulting in an immunosuppressive microenvironment (24). This enables tumor cells to evade immune surveillance via VEGF, a key mediator that reduces tumor infiltration by T cells, and increases the number and proliferation of immunosuppressive cells such as regulatory T cells, myeloid-derived suppressor cells, and M2-like tumor-associated macrophages (25, 26). In turn, PD-L1 expression can inhibit T-cell activation and prevent an innate cytotoxic T-cell response against tumors (27). In many solid tumors, these well-recognized events contribute to angiogenesis and growth. Atezolizumab, a PD-L1 inhibitor, can suppress immunosuppressive cells and indirectly downregulate the expression of angiogenic factors (28). However, due to the unstable expression of PD-L1 (29), insufficient lymphocyte infiltration in SCLC (30), and rapid disease progression, the efficacy of immunotherapy may be compromised.

Notably, anlotinib may boost the efficacy of immunotherapy by increasing the number of innate immune cells, preventing exhaustion of CD4+T cells, and reducing PD-L1expression via inactivation of the AKT pathway in vascular endothelial cells (31, 32). Thus, a combination of anlotinib with a PD-L1 inhibitor appears to transform the tumor microenvironment into an immune-permissive status, also enhancing the synergistic efficacy of the antitumor response by suppressing tumor neovascularization (33). Indeed, the results of a cohort study suggested that infiltration of immune cells, such as CD3+ T cells, CD4+ T cells, and monocytes, strongly influences long-term survival in SCLC (34). Supporting this hypothesis, the lymphocyte counts in the tumor immune microenvironment of the ES-SCLC patient in this case report remained stable throughout the course of treatment.

The most common treatment-related adverse events reported for the combination of anlotinib with ICIs in SCLC are hypertension, hepatic dysfunction, hypothyroidism, anorexia, fatigue, oral ulcers, hand-foot syndrome, diarrhea, and bleeding (10, 11). These adverse events are manageable and well-tolerated, with no treatment-related deaths reported. Asthma during immunotherapy is rare and has only been reported during treatment with the PD-1 inhibitor nivolumab in male patients with non-small-cell lung cancer (NSCLC) (35, 36). To our knowledge, this is the first report of an asthma-negative SCLC patient who developed asthma after treatment with atezolizumab plus anlotinib. The binding of PD-1 to its ligands, PD-L1 and PD-L2, is closely related to the increase in CD4+ T helper type 2 (Th2) lymphocytes and IgE-dependent activation in allergic diseases. CD4+ T cells are predominantly associated with allergic asthma and enhanced eosinophil activity, contributing to airway hyperreactivity (AHR) and cytokine secretion (37). Th2 cells are considered crucial in AHR because they produce IL-4 and IL-13 to induce an increase in IgE production (38). PD-L1, a negative regulator of T cells, strongly stimulates PD-1 expression after antigen presentation, leading to CD4+ T-cell exhaustion and tolerance (39, 40). In a murine model, PD-L1 favored Th2-driven inflammation by upregulating IL-4 and downregulating IFN-γ, which seems crucial for increasing AHR (41). However, in a human asthma model, significantly downregulated PD-L1 expression was observed in dendritic cells (DCs) by circulating CD4+ T cells, along with high IgE concentrations detected in patients with allergic asthma (42). The basis of this discrepancy between humans and mice is unclear and may be related to species differences, model sensitization, or disease progression. Importantly, regulation of the PD-1/PD-L1 pathway by atezolizumab can lead to Th2-mediated eosinophil activation through a type-2 innate lymphoid cell-dependent mechanism (43). In this regard, eosinophilia has been proposed as a prognostic and potentially predictive biomarker for patients with lung cancer receiving immunotherapy and is significantly associated with an increased chance of achieving disease control and a higher probability of treatment toxicity (44). Current clinical data suggest that increased blood eosinophil counts may reflect favorable outcomes in patients treated with ICIs for advanced lung cancer. Nevertheless, more clinical trials are needed to further elucidate the value of eosinophilia as a prognostic biomarker and the correlation between treatment response and toxicity (45). Notably, a previous study indicated that AHR may be acquired by high-risk factors such as cigarette smoking, squamous cell lung cancer, and peripheral blood eosinophilia (46). Although the patient had no history of asthma, eosinophilia, or smoking before treatment, she experienced dyspnea with wheezing 10 months after starting the treatment for ES-SCLC. The eosinophil counts and IgE levels were significantly increased, possibly due to the administration of atezolizumab, which restored allergic inflammation and the tumor microenvironment. Based on these results and considering the above symptoms, radiographic findings, and lung function tests, ICI-related asthma was ultimately diagnosed. This would thus suggest a dual effect of PD-L1 blockade, involving therapeutic effects on SCLC, and potential activity as an asthmagen. Allergic inflammation usually occurs 2–12 months after ICI treatment (47). In this regard, ICI-related changes in immune tolerance in the tumor microenvironment might affect airway tolerance, leading to the occurrence of AHR. Hence, potential markers such as TGF-β, IL-10, and IL-17A need to be monitored in patients receiving ICIs (48). To develop preventive and control measures, further investigation of the specific mechanisms by which immunotherapeutic modulation of PD-L1 influences airway inflammation in SCLC is required.

In reviewing the treatment course of the ES-SCLC patient, we observed long and high-quality survival after a combined treatment with atezolizumab and anlotinib. Furthermore, this patient benefited from a PFS of 22 months with a currently stable disease status. ICI-related asthma after PD-L1 blockade in patients with SCLC has rarely been reported. This adverse event has not been reported during combination treatments in other SCLC cases.

## Conclusion

The combination of atezolizumab and anlotinib appears to be a potentially effective therapy for ES-SCLC, possibly achieving long-lasting disease control and improved survival when closely monitored. The unusual occurrence of treatment-related adverse events should be carefully monitored and timely addressed to enable providing theoretical support. Clinical verification in the setting of adequately powered clinical trials and the assessment of adverse events are necessary to confirm the efficacy and safety of this combination therapy in ES-SCLC.

## Data availability statement

The original contributions presented in the study are included in the article/supplementary material. Further inquiries can be directed to the corresponding author.

## Ethics statement

The studies involving humans were approved by Second Affiliated Hospital of Chongqing Medical University. The studies were conducted in accordance with the local legislation and institutional requirements. Written informed consent for participation was not required from the participants or the participants’ legal guardians/next of kin in accordance with the national legislation and institutional requirements. Written informed consent was obtained from the individual(s) for the publication of any potentially identifiable images or data included in this article.

## Author contributions

WD: Conceptualization, Funding acquisition, Writing – original draft. JC: Data curation, Writing – review & editing. X-YD: Data curation, Writing – review & editing.
